# Revision surgery for instrumentation failure after total en bloc spondylectomy: a retrospective case series

**DOI:** 10.1186/s12891-020-03622-6

**Published:** 2020-09-02

**Authors:** Kazuya Shinmura, Satoshi Kato, Satoru Demura, Noriaki Yokogawa, Noritaka Yonezawa, Takaki Shimizu, Norihiro Oku, Ryo Kitagawa, Makoto Handa, Ryohei Annen, Hideki Murakami, Hiroyuki Tsuchiya

**Affiliations:** 1 Department of Orthopedic Surgery, Graduate School of Medical Sciences, Kanazawa University, 13-1 Takara-machi, Kanazawa, 920-0961 Japan; 2 Department of Orthopedic Surgery, Nagoya City University Graduate School of Medical Sciences, Nagoya, Japan

**Keywords:** Total en bloc spondylectomy, Instrumentation failure, Revision surgery, Cobalt chromium, Bone fusion, Liquid nitrogen

## Abstract

**Background:**

There have been several reports of instrumentation failure after three-column resections such as total en bloc spondylectomy (TES) for spinal tumors; however, clinical outcomes of revision surgery for instrumentation failure after TES are seldom reported. Therefore, this study assessed the clinical outcomes of revision surgery for instrumentation failure after TES.

**Methods:**

This study employed a retrospective case series in a single center and included 61 patients with spinal tumors who underwent TES between 2010 and 2015 and were followed up for > 2 years. Instrumentation failure rate, back pain, neurological deterioration, ambulatory status, operation time, blood loss, complications, bone fusion after revision surgery, and re-instrumentation failure were assessed. Data were collected on back pain, neurological deterioration, ambulatory status, and management for patients with instrumentation failure, and we documented radiological bone fusion and re-instrumentation failure in cases followed up for > 2 years after revision surgery.

**Results:**

Of the 61 patients, 26 (42.6%) experienced instrumentation failure at an average of 32 (range, 11–92) months after TES. Of these, 23 underwent revision surgery. The average operation time and intraoperative blood loss were 204 min and 97 ml, respectively. Including the six patients who were unable to walk after instrumentation failure, all patients were able to walk after revision surgery. Perioperative complications of reoperation were surgical site infection (*n* = 2) and delayed wound healing (*n* = 1). At the final follow-up, bone fusion was observed in all patients. No re-instrumentation failure was recorded.

**Conclusion:**

Bone fusion was achieved by revision surgery using the posterior approach alone.

## Background

Total en bloc spondylectomy (TES) was developed for complete surgical resection for spinal tumors [1, 2]. TES was reported to produce less local recurrence and better survival than piecemeal resection for primary tumors [3–7], aggressive benign tumors [1, 8], or metastatic spinal tumors [1, 9]. TES involves complete resection of the affected vertebra(e) and surrounding musculoligamentous supportive tissues. The surgery presents a challenge for spinal reconstruction owing to the complete discontinuity in the spinal column. To restore spinal stability after resection, robust instrumentation and bone grafting are needed, with anterior column support [10–12]. Because TES is performed in patients with a relatively good prognosis [13], long-term stabilization of the spine is important. Failure of bone fusion can cause instrumentation failure, causing back pain, neurological deterioration, and decreased performance of activities of daily living (ADL) [13–18]. In most cases, revision surgery is required to improve spinal stability and symptoms.

Since 2010, instead of harvesting autografts from the ilium or fibula, the resected lamina and vertebral body from the TES are frozen in liquid nitrogen and used as grafted bone for spinal reconstruction. This technique has the following benefits: no pain at the bone harvest site, shortened operative time, decreased blood loss, and additional antitumor immune response [19]. In this procedure, the strategy of revision surgery is more important than that in the conventional procedure because the instrumentation failure rate may increase with a decreased bone fusion rate.

Despite the fact that several studies have examined instrumentation failure after TES [11–14], there are few published reports on revision surgery for instrumentation failure after TES. Therefore, the aims of this study were to determine the rate of instrumentation failure and to document clinical outcomes of revision surgery for instrumentation failure after TES with reconstruction using frozen autografts treated with liquid nitrogen.

## Methods

### Study population

This retrospective study was approved by our institutional review board, and informed consent was obtained from all patients. Between 2010 and 2015, 114 patients with primary or metastatic spinal tumors underwent TES at our institute. Of these, 33 patients died within 2 years, and 20 patients were lost to follow-up. Finally, 61 patients (53.5%) who could be followed up for > 2 years after TES were included and comprised 35 men and 26 women, with a mean age of 52.6 (range 14–73) years. The mean follow-up period was 60 (range 24–101) months. Of the 61 patients, 10 had primary malignant tumors, 10 had primary aggressive benign tumors, and 41 had metastatic tumors. In 41 patients with metastatic disease, metastases were diagnosed as having oligometastatic cancer. The thoracic, thoracolumbar, and lumbar spines were affected in 27, 27, and seven patients, respectively.

### Surgical procedures

Primary surgery (TES) consisted of en bloc laminectomy after transpedicular osteotomy, subsequent en bloc corpectomy, and spinal reconstruction [8, 20]. After en bloc laminectomy, two-above and two-below segmental fixations were performed in all patients. Two rods were used in all patients. Titanium alloy rods (diameter: 5.5 mm) were used in 59 patients. Cobalt chromium rods (diameter: 6.0 mm) were used in two patients. No hooks or wires were used. After posterior instrumentation, anterior reconstruction was performed using a titanium mesh cage (MOSS-Miami; DePuy Motech, Warsaw, IN, USA) filled with frozen autografts treated with liquid nitrogen. To increase spinal stability, the posterior instrumentation was adjusted to slightly compress the inserted vertebral cage. Finally, at least two transverse connectors were applied. In all cases, a rigid spinal brace was used for a postoperative period of 3 months, followed by a soft brace for another 3 months.

**Fig. 1:**
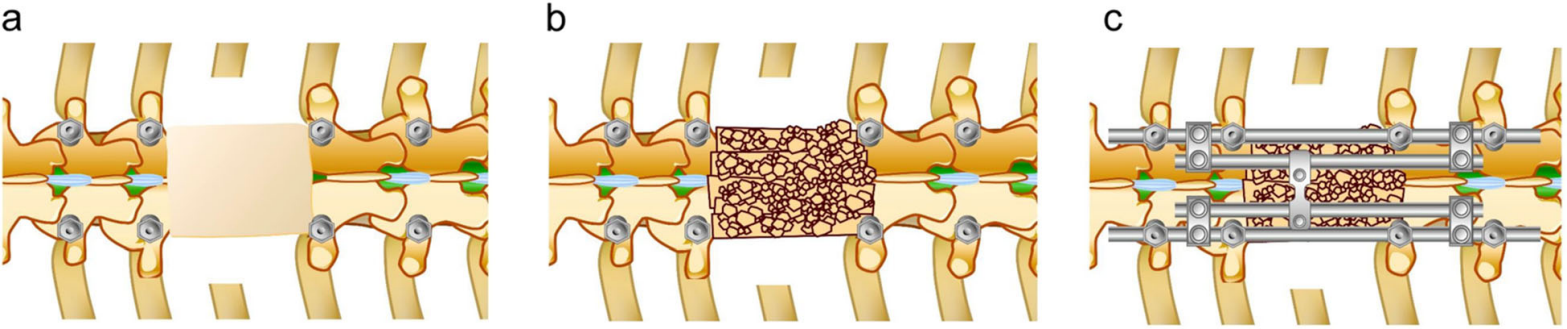
Schema of revision surgery after instrumentation failure. **a** The scar tissue of the resected vertebral area and the adjacent laminae are exposed. **b** Fresh autologous bone strut graft harvested from the iliac crest is placed on the scar tissue of the resected vertebral area to form a bridge between the adjacent laminae. **c** To increase stability, additional rods are applied

Revision surgery after instrumentation failure was performed via the posterior approach alone in all cases. Broken rods and broken or loosening pedicle screws were replaced. In some patients, segmental fixation was extended to increase stability. In most patients, a fresh autologous bone strut graft harvested from the iliac crest was placed on the scar tissue of the resected vertebral area to form a bridge between the adjacent laminae (Fig. 1a, b). In some patients, additional fresh autologous bone grafts were placed around the cage. The posterior instrumentation was adjusted to slightly compress the inserted vertebral cage. To increase stability, four rod fixations were applied in most cases (Fig. 1c). No patients had cage replacement via the anterior approach. In all cases, a rigid spinal brace was used for a postoperative period of 3 months, followed by a soft brace for another 3 months.

### Evaluation

Instrumentation failure was determined based on follow-up plain radiographs. In cases of instrumentation failure, computed tomography (CT) was performed to determine the details of instrumentation failure. Back pain, neurological deterioration, ambulatory status, and management (conservative or revision surgery) were recorded. Operative time, intraoperative blood loss, and complications of the revision surgery were documented. Finally, we evaluated the extent of radiological bone fusion using multiplanar reconstruction CT in cases of re-instrumentation failure that could be followed up for > 2 years after revision surgery. The graft bone in the cage was continuous with the upper and lower adjacent vertebral bodies, and a cage filled with bone tissue was considered bone fusion within the cage.

## Results

Of the 61 patients, 26 (42.6%) experienced instrumentation failure at an average of 32 (range, 11–92) months after TES. The mean body mass index of the patients who experienced instrumentation failure was 23.4 (range, 16.4–32.3). Of the 26 patients who experienced instrumentation failure, 4 (15.3%) underwent radiation therapy for spinal tumors before TES. Ten patients had primary tumors, comprising giant cell tumor in 3 patients, chondrosarcoma in 1, epithelioid sarcoma in 1, liposarcoma in 1, Ewing’s sarcoma in 1, synovial sarcoma in 1, chordoma in 1 and aggressive hemangioma in 1. Sixteen patients had metastatic tumors from renal cell carcinoma in 4 patients, breast cancer in 3, thyroid cancer in 2, lung cancer in 2, hepatocellular carcinoma in 1, esophageal cancer in 1, parotid cancer in 1, leiomyosarcoma in 1, unknown primary squamous cell carcinoma in 1. Rod breakage was observed in 20 patients, screw breakage in two patients, rod and cage breakage in two patients, cage breakage in one patient, and screw and cage breakage in one patient. However, no evidence of local recurrence was found at the surgical site throughout the follow-up period in any patient. Of the 26 patients, 19 (73.1%) experienced back pain, 8 (30.8%) had lower-extremity neurological deterioration, and 6 (23.1%) were unable to walk because of these symptoms (Table 1). Of the 26 patients, 23 underwent revision surgery and three did not undergo revision surgery. In patients without revision surgery, one patient (patient 26; Table 1) with an asymptomatic rod breakage died from cancer 1 year after instrumentation failure. Patients with asymptomatic cage and screw breakage (patients 24 and 25; Table 1) survived for 1 year and 3 years, respectively. Neither experienced advanced spinal instability nor related symptoms. Revision surgery was performed with posterior instrumentation replacement in all 23 patients, posterior bone grafting in 21 patients, and additional bone grafting around the cage in three patients. To increase stability, the procedure was extended by three segmental fixations above and below in three patients, two segmental fixations above and below in one patient, and two segmental fixations above and four segmental fixations below in two patients. All of the six patients with extended fixation had screw loosening. In the remaining cases without screw loosening, instrumentation was not extended. Two rods were used in four patients, three rods were used in one patient, and four rods were used in 18 patients. Titanium alloy rods (diameter: 5.5 mm) and cobalt chromium rods (diameter: 6.0 mm) were used in 4 and 20 patients, respectively. The average operative time and intraoperative blood loss were 207 ± 45 (range, 121–287) min and 93 ± 81 (range, 20–270) ml, respectively. Intraoperative findings via a posterior approach did not reveal any obvious pseudoarthroses in the patients because the scar tissue covered the anterior column where the pseudoarthroses existed, but mild metallosis at the rod fracture site was observed in nine patients. All patients, including the six patients who were unable to walk due to symptomatic instrumentation failure, maintained or recovered their ambulation function after revision surgery. Perioperative complications of revision surgery were superficial surgical site infection in two patients and delayed wound healing in one patient. They were successfully treated with conservative therapies using antibiotics and basic fibroblast growth factor spray.

**Table 1:** Details of patients who experienced instrumentation failure

No.	Age	Sex	BMI	Type of tumor	RT	Resection level	Broken parts in instrumentation failure	Duration after TES (mo)	Back pain	Neurological deterioration	Ambulatory	Revision surgery
1	39	M	24.0	thyroid cancer	no	T4	2 rods	42	+	–	yes	yes
2	63	F	21.0	thyroid cancer	no	T4, 5, 6	1 rod	74	+	–	yes	yes
3	57	F	22.3	parotid cancer	no	T5, 6	2 rods	92	+	–	yes	yes
4	59	F	22.6	breast cancer	yes	T6, 7	2 rods	24	–	bilateral leg weakness	no	yes
5	68	M	22.4	hepatocellular carcinoma	yes	T7	2 rods	51	–	bilateral leg weakness	yes	yes
6	24	M	22.0	leiomyosarcoma	no	T7,8	1 rod	29	+	–	yes	yes
7	41	F	16.4	synovial sarcoma	yes	T8, 9	2 rods	37	+	–	yes	yes
8	68	M	23.2	renal cell carcinoma	no	T8, 9	2 rods	18	+	–	yes	yes
9	59	M	24.2	renal cell carcinoma	no	T8, 9, 10	2 rods	11	+	–	no	yes
10	59	M	24.3	lung cancer	no	T8,9, 10	2 rods and cage	25	+	bilateral leg weakness	no	yes
11	66	F	18.4	esophageal cancer	no	T9	1 rod	17	+	–	yes	yes
12	26	M	27.0	epithelioid sarcoma	no	T10	2 rods	34	+	bilateral leg paresthesia	yes	yes
13	40	M	26.3	liposarcoma	no	T10	1 rod	38	–	–	yes	yes
14	45	M	23.6	chondrosarcoma	no	T10	1 rod	32	+	–	yes	yes
15	70	M	25.2	unknown	no	T10, 11	2 rods	11	+	–	no	yes
16	14	M	24.3	Ewing’s sarcoma	no	T12	2 screws	22	–	–	yes	yes
17	48	M	28.4	renal cell carcinoma	no	T12, L1	2 rods	19	+	bilateral leg paresthesia	yes	yes
18	66	F	22.4	breast cancer	no	L1	1 rod	18	+	–	yes	yes
19	16	M	22.8	chordoma	no	L1, 2	2 rods and cage	21	+	–	yes	yes
20	64	M	19.9	aggressive hemangioma	no	L2	1 rod	23	+	–	yes	yes
21	49	F	30.9	giant cell tumor	no	L4,5	2 rods	39	+	bilateral leg paresthesia	no	yes
22	25	F	17.2	giant cell tumor	no	L4	cage and 1 screw	31	+	bilateral leg paresthesia	yes	yes
23	50	M	32.3	lung cancer	no	L5	2 rods	24	+	bilateral leg pain	no	yes
24	21	F	24.3	giant cell tumor	no	T11	cage	24	–	–	yes	no
25	41	F	22.2	breast cancer	no	L3	2 screws	35	–	–	yes	no
26	70	M	20.8	renal cell carcinoma	yes	L3	1 rod	30	–	–	yes	no

*BMI* body mass index, *RT* radiation therapy

Eighteen patients could be followed up for > 2 years after revision surgery (average follow-up period: 41 months) by radiological evaluation. At the last follow-up, bone fusion was achieved within the anterior cage and at the posterior bone graft in 11 patients, within the anterior cage in two patients, and at the posterior bone graft in five patients. There was no re-instrumentation failure after revision surgery (Table 2).

**Table 2:** Details of patients who underwent revision surgery

No.	Resection level	Operation time (min)	bleeding (ml)	The number of rod	Composition of rod	Extended segmental fixation	Bone graft	Complications	Duration after revision surgery (mo)	Bone fusion	Re-instrumentation failure
1	T4	240	50	2	Ti	–	posterior	–	58	anterior and posterior	no
2	T4, 5, 6	214	40	4	Cocr	–	posterior	–	22	anterior and posterior^a^	no
3	T5, 6	173	120	4	Cocr	–	posterior	–	9	unachieved^a^	no
4	T6, 7	248	20	4	Cocr	3-above 3-below	–	–	28	anterior	no
5	T7	222	50	3	Cocr	2-above 4-below	posterior	delayed wound healing	17	posterior^a^	no
6	T7,8	213	110	2	Cocr	–	–	–	31	anterior	no
7	T8, 9	208	250	4	Ti	–	posterior	–	32	anterior and posterior	no
8	T8, 9	132	20	4	Cocr	3-above 3-below	posterior	–	13	unachieved^a^	no
9	T8, 9, 10	121	120	4	Cocr	–	posterior	–	44	anterior and posterior	no
10	T8,9, 10	239	130	4	Cocr	3-above 3-below	posterior and anterior	–	38	anterior and posterior	no
11	T9	164	50	4	Cocr	–	posterior	SSI	35	anterior and posterior	no
12	T10	168	130	4	Cocr	–	posterior	–	32	posterior	no
13	T10	150	20	4	Cocr	–	posterior	–	37	anterior and posterior	no
14	T10	156	30	4	Cocr	–	posterior	–	40	posterior	no
15	T10, 11	235	270	2	Ti	2-above 4-below	posterior and anterior	–	5	unachieved^a^	no
16	T12	224	30	4	Cocr	2-above 3-below	posterior	–	28	anterior and posterior	no
17	T12, L1	203	20	4	Cocr	–	posterior	–	36	anterior and posterior	no
18	L1	143	50	4	Cocr	–	posterior	–	42	anterior and posterior	no
19	L1, 2	287	80	4	Cocr	–	posterior and anterior	–	52	anterior and posterior	no
20	L2	216	50	4	Cocr	–	posterior	–	46	anterior and posterior	no
21	L4,5	262	115	4	Cocr	–	posterior	SSI	49	posterior	no
22	L4	241	220	2	Ti	–	posterior	–	60	posterior	no
23	L5	239	250	4	Cocr	–	posterior	–	56	posterior	no

*Cocr* cobalt chromium, *Ti* titanium alloy, *SSI* surgical site infection

^a^follow up period after revision surgery was 2 years or less

### Illustrative case (case 18)

**Fig. 2:**
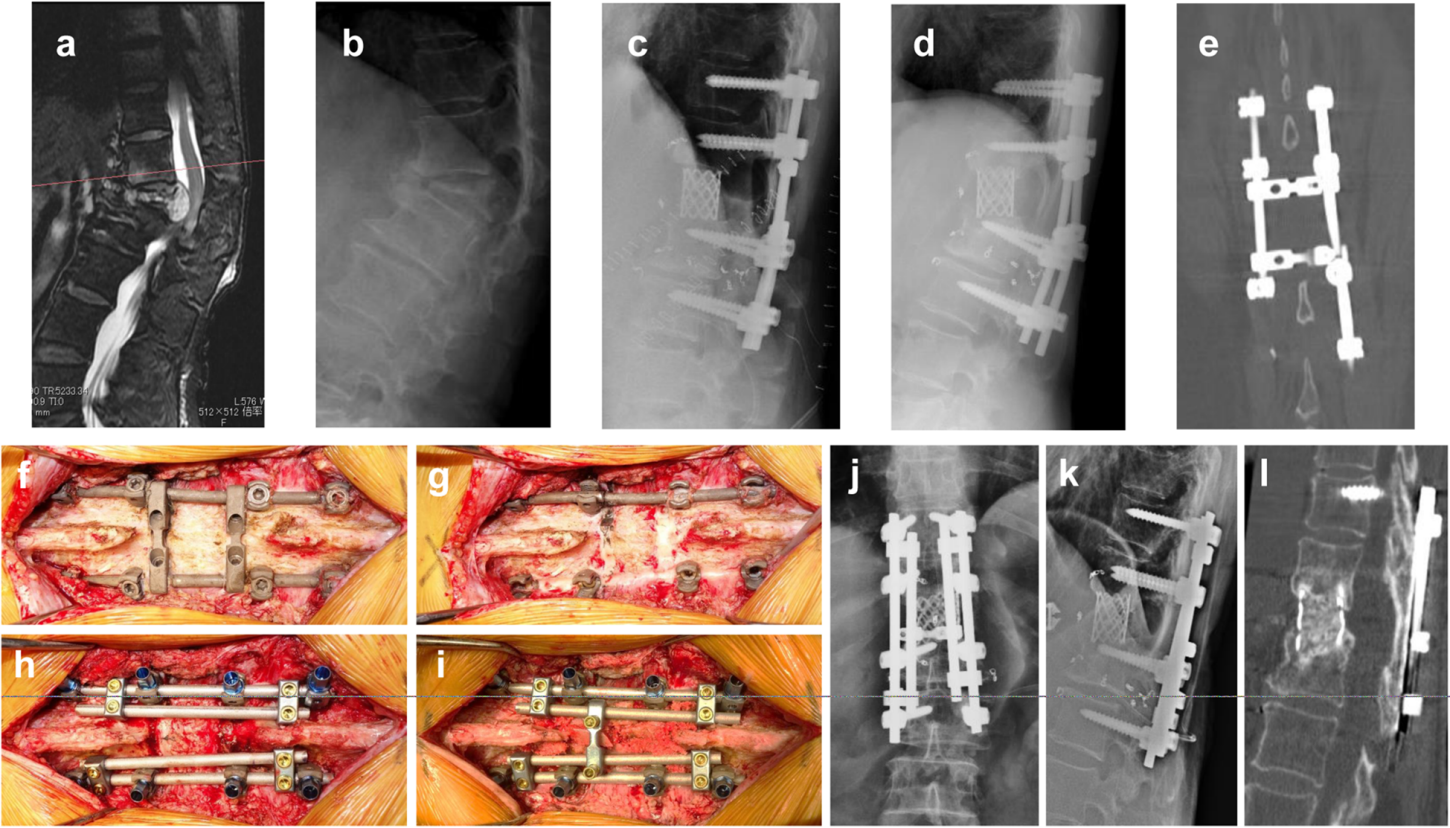
Case 18. A 66-year-old woman with breast cancer metastasis at L1. **a** Preoperative sagittal T2 magnetic resonance image revealing tumor involvement at L1. **b** Lateral radiograph revealing pathological fracture at L1. **c** Lateral radiograph after total en bloc spondylectomy. **d** Radiographs at 18 months after surgery revealing increased local kyphosis angle between T12 and L2. **e** Coronal computed tomography (CT) scan revealing breakage of the left rod. **f** Posterior instrumentation is exposed using the previous midline incision. **g** The left rod is broken at the area proximal to the L2 pedicle screw. **h** Loosening pedicle screw at the left T11 level is replaced, and four cobalt chromium rods are inserted. **i** Autologous bone graft is placed on adjacent T12 and L2 lamina and scar tissue of the resected L1 lamina area. **j**, **k** Radiograph after revision surgery. **l** Sagittal CT scan at 42 months after revision surgery revealing bone fusion within the cage and at the posterior bone graft

The patient was a 66-year-old woman with a solitary spinal metastasis from breast cancer. She underwent TES of L1 with two-above and two-below segmental fixation using two titanium alloy rods. At 18 months after TES, radiography revealed an increased local kyphosis angle between T12 and L2. Breakage of the left rod was noted on CT. Revision surgery was performed using a single posterior approach. The rod on the left side was broken at the proximal level of the L2 pedicle screw. Transverse connectors and bilateral rods were removed, and the loosening pedicle screw at the left T11 level was replaced. Spinal fixation was performed using four cobalt chromium rods (diameter: 6.0 mm). An autologous strut bone graft was placed on the scar tissue of the resected L1 lamina area and the adjacent T12 and L2 laminae. The posterior instrumentation was adjusted to slightly compress the inserted vertebral cage. No perioperative complications occurred. The patient re-acquired ambulatory function, without aid, 1 week after the revision surgery. At 42 months after the revision surgery, bone fusion within the cage and at the posterior bone graft was observed on CT (Fig. 2).

## Discussion

We reported the instrumentation failure rate of patients with instrumentation failure after TES with reconstruction using frozen autografts treated with liquid nitrogen. Revision surgery was performed using the posterior approach alone. Bone fusion was achieved, and there was no re-instrumentation failure in any patient at the follow-up period > 2 years.

With continuing advances in cancer therapy, acceptable long-term prognosis can be expected even in patients with metastatic spinal tumors [21–24]. In TES, en bloc resection of a tumor-bearing vertebra can be curative, leading to longer-term survival, and achieving bone fusion of the reconstructed vertebral body is essential [25]. However, instrumentation failure caused by unsuccessful bone fusion is not a rare complication. Park et al. [10] reported that 12 (37.5%) of 32 patients experienced rod breakage at an average of 29.2 (range, 8–93) months after TES. Sciubba et al. [14] reported instrumentation failure in 9 (39.1%) of 23 patients who underwent lumbar-spine TES. Matsumoto et al. [12] reported instrumentation failure in 6 (40%) of 15 patients who underwent TES. In our study, instrumentation failure following the TES procedure was identified in 26 (42.6%) of 61 patients at an average of 32 (range, 11–92) months after TES, which was comparable to that of other studies.

The previously reported incidence rate of instrumentation failure after TES using the same reconstruction method as ours (except using fresh autologous bone for bone grafting) was 17.0% (8/47) [11]; in the present study, the instrumentation failure rate after TES using frozen bone was 42.6% (26/61). It was reported that bone formation tended to be delayed when frozen bone autografts were used compared with fresh bone autografts [25]; therefore, the instrumentation failure rate in the present study was higher than that previously reported [11]. Although bone fusion was delayed, complete bone fusion within the cage was obtained in the TES model canine using frozen bone [25]. The instrumentation failure rate following the first procedure was higher in the present study; however, stability was maintained for a long time in 35 (57.4%) of 61 patients. Considering the advantages of using liquid nitrogen-treated bone, we continue using frozen bone autografts in spinal reconstruction during TES. To decrease the incidence of instrumentation failure, we recently began using a more robust cage and cobalt chrome rods to create a stiffer construct of the operated spine and having additional bone graft around the cage to facilitate bone fusion.

In the present study, back pain and neurological deterioration caused by instrumentation failure developed in 19 (76.1%) and 8 (30.8%) patients, respectively. Matsumoto et al. [12] reported that all 6 (100%) patients experienced back pain and 1 (16.7%) experienced neurological deterioration at the time of instrumentation failure. Park et al. [10] reported that back pain developed in 7 (58.3%) patients, and no patients had neurological deterioration at the time of instrumentation failure. These findings suggest that most patients with instrumentation failure experienced significant clinical symptoms. Revision surgery is necessary to prevent decreased ADL performance among symptomatic patients with instrumentation failure.

Instrumentation failure is caused by delayed union between the cage and the vertebral body [11], and revision surgery is performed to achieve robust restabilization and bone fusion. In the present study, we performed robust restabilization in most patients by replacing titanium rods with cobalt chromium rods and by increasing the number of rods. We also performed bone grafting at the posterior aspect of the spine. During the primary surgery, bone grafting at the posterior element was difficult because there was no bed for bone grafting at the level of the resected vertebra. Moreover, because the space was covered with scar tissue, bone grafting was straightforward and secured during the revision surgery.

In the present study, 13 (72.2%) of 18 patients with > 2 years of follow-up after revision surgery achieved bone fusion within the cage, but the remaining 5 (27.8%) did not achieve bone fusion; nevertheless, bone resorption within the cage improved. We believe that robust restabilization of the posterior instrumentation increased the stability of the spine, which facilitated bone fusion within the cage. In addition, bone fusion at the posterior aspect of the spine, which could not be applied in the primary surgery without the scar tissue of the resected vertebral area, was achieved earlier than that within the cage in cases where posterior bone grafting was performed. We believe that attaining bone fusion at the posterior aspect further increased the stability of the spine and favored bone fusion within the cage. This finding indicates that replacement of the cage using the anterior approach is unnecessary when the cage is restabilized using a stiffer construction by exchange and supplement of posterior instruments. Our recommended revision procedure is to perform posterior restabilization with three or four cobalt chromium rods and posterior bone grafting. In cases with a severely damaged cage (not observed in this study cohort), cage replacement using the anterior approach should be considered.

This study has some limitations. The small and heterogenous cohort with several tumor histologies and adjuvant therapies and the retrospective manner of data collection can introduce bias and errors. This retrospective study included various reconstruction procedures (e,g, the material and number of rods in revision surgery) differed depending on the time of surgery. The follow-up time was limited as well. Longer follow-up is required to determine the accurate incidence of instrumentation failure after revision surgery. Although all patients were diagnosed as having oligometastatic cancer in the present study, the indications for TES remain controversial because less invasive surgeries (e.g. separation surgery) have shown significant results recently. Despite these limitations, this study demonstrated the rate of instrumentation failure after TES with reconstruction using frozen autografts treated by liquid nitrogen and described a relatively simple and effective strategy for revision surgery and its favorable outcomes. The results obtained from this study will contribute to revision surgery for instrumentation failure after TES.

## Conclusions

In patients with instrumentation failure after TES, posterior instrumentation replacement and posterior bone grafting were performed during revision surgery. In this study, bone fusion was observed in all patients with > 2 years of follow-up after revision surgery, and no re-instrumentation failure was observed during follow-up. Our findings suggest that bone fusion can be achieved by revision surgery using the posterior approach.

## Data Availability

The datasets generated during and/or analyzed during the current study are available from the corresponding author on reasonable request.

## Abbreviations

ADL: Activities of daily living
CT: Computed tomography
TES: Total en bloc spondylectomy
